# Adsorptive Separation of CO_2_ by a Hydrophobic Carborane-Based Metal–Organic Framework under Humid Conditions

**DOI:** 10.1021/acsami.2c20373

**Published:** 2023-01-24

**Authors:** Lei Gan, Eduardo Andres-Garcia, Guillermo Mínguez Espallargas, José Giner Planas

**Affiliations:** † Institut de Ciència de Materials de Barcelona (ICMAB-CSIC), Campus UAB, 08193Bellaterra, Spain; ‡ Instituto de Ciencia Molecular (ICMol), Universidad de Valencia, c/Catedrático José Beltrán, 2, 46980Paterna, Spain

**Keywords:** metal−organic frameworks, carboranes, hydrophobic, selective gas separation, breakthrough, humid conditions, CO_2_ adsorption

## Abstract

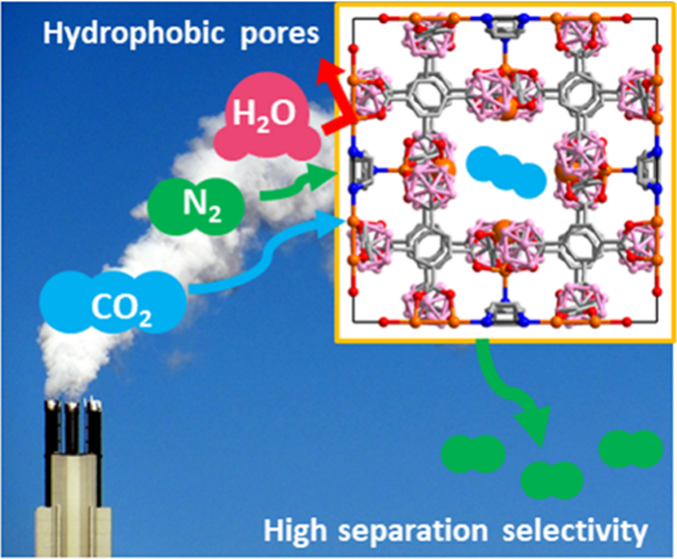

We report that the carborane-based metal–organic framework (MOF) ***m*****CB-MOF-1** can achieve high adsorptive selectivity for CO_2_:N_2_ mixtures. This hydrophobic MOF presenting open metal sites shows high CO_2_ adsorption capacity and remarkable selectivity values that are maintained even under extremely humid conditions. The comparison of ***m*****CB-MOF-1'** with MOF-74(Ni) demonstrates the superior performance of the former under challenging moisture operation conditions.

## Introduction

The global greenhouse effect, which is mostly caused by the emission of CO_2_ into the atmosphere, has attracted more and more attention.^1^ Apart from natural processes, the consumption of carbon-based fossil fuels from power plants is mainly responsible for generating this anthropogenic CO_2_ emission.^2,3^ Therefore, carbon capture and storage (CCS) from the postcombustion flue gas is a wise method to reduce CO_2_ environmental impact. So far, a number of technologies and materials have been developed for CO_2_ capture and separation, such as aqueous ammonia and amine-functionalized solid adsorption,^4−6^ membrane separation,^7,8^ cryogenics distillation,^9−11^ among others. Compared with traditional techniques, adsorption-based methods using porous materials to capture or separate CO_2_ with less energy consumption and cost shows great advantages among these technologies. Activated carbon, zeolites, and carbon molecular sieves have been extensively studied as adsorbents for CO_2_ separation.^12−14^ These materials present several drawbacks such as difficult regeneration procedures and, quite importantly, poor tunability with the procedure conditions. Thus, developing economical and highly regenerative materials to efficiently capture and separate CO_2_ from flue gas is highly desirable.

Metal–organic frameworks (MOFs) exhibit outstanding separation performances toward diverse binary gas mixtures due to their large surface areas, tunable pore size and pore surface, and existing open metal sites.^15−18^ Many MOFs have been reported to efficiently separate CO_2_:N_2_ mixtures under dry conditions; however, their performance cannot be often maintained under humid conditions.^19,20^ The reason for such a decrease in performance can be twofold. On one hand, there are MOFs that are unstable upon exposure to water and thus cannot be used in gas separation under humid conditions. This is the case, for example, of two of the most well-known adsorbents, HKUST-1 ([Cu_3_(BTC)_2_]_*n*_, BTC^3–^ = benzene-1,3,5-tricarboxylate)^21^ and MOF-5 (Zn_4_O(BDC)_3_, BDC^2–^ = 1,4-benzodicarboxylate).^22^ The reason for such reduced separation performance under humid conditions is the collapse of the framework by slow hydrolysis when exposed to moisture conditions, even to limited amounts of water. On the other hand, there are other MOFs built by a robust metal cluster that are water-stable, but a common decrease of CO_2_ capacity and selectivity is also observed under humid conditions.^23−26^ For instance, CO_2_ adsorption capacity on UiO-66-NH_2_ decreased 88% at 70% RH compared to that at dry conditions.^10^ Considering that flue gas contains 5–7% water, these MOFs can only be used for CO_2_ separation after a dehydration pretreatment of the gas stream, certainly increasing the economic costs and energy demand of the separation process. Consequently, new approaches are being investigated to increase the robustness of these MOFs absorbents and maintain their water stability, such as alkylamine grafting^27^ or PEI composite impregnation.^28^ In addition, functionalizing the original linker using hydrophobic functional groups such as fluorine or alkyl groups offers an opportunity to improve the hydrolytic stabilities and repel competitive water molecules from entering into MOFs. However, this methodology requires a more complicated organic synthesis reaction for obtaining the functionalized organic linker.^29−31^ Given that water vapor is inevitable among most industrial flue gas mixtures, the screening of facile synthesizable water-stable MOFs for efficient CO_2_ separation under humid conditions is both essential and challenging.

**Figure 1:**
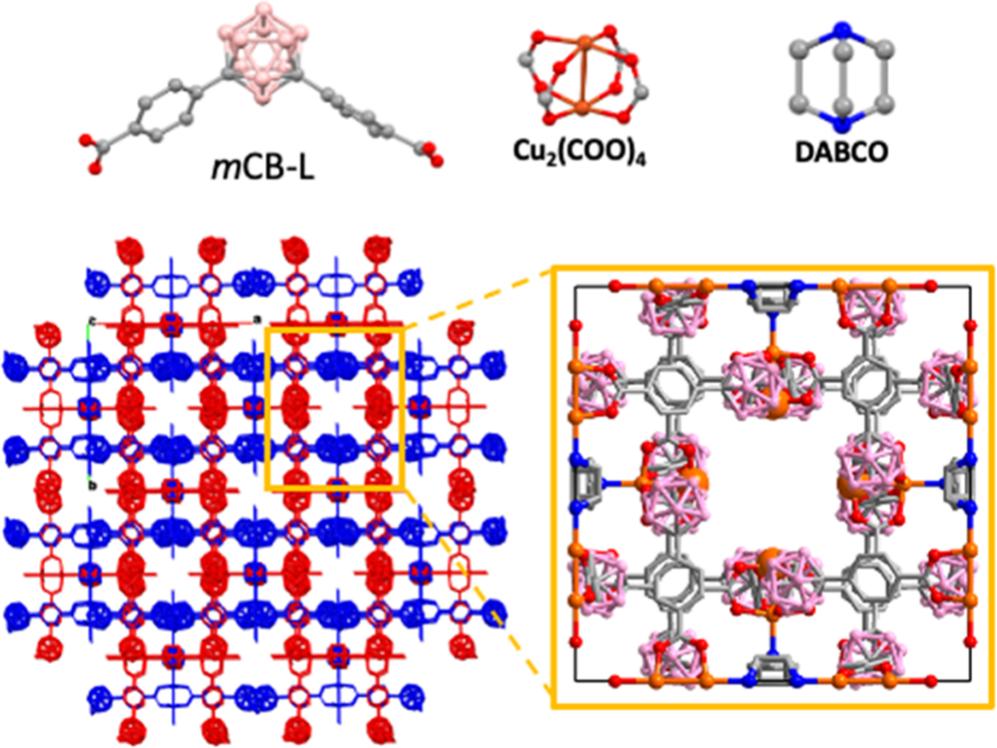
Representation of building units (top) and 3D structure (bottom) of ***m*CB-MOF-1**. Interpenetrated networks are colored blue and red for clarity. The yellow square shows the detail for the pore details along the *c*-axis showing the environment of four Cu_2_ paddle wheel units (enlarged orange spheres) surrounded by carborane moieties. Color codes: Cu, orange; B, pink; C, gray; N, blue; O, red. H atoms are omitted for clarity.

Icosahedral carboranes 1,*n*-C_2_B_10_H_12_ (*n* = 2 (*ortho* or *o*), 7 (*meta* or *m*), or 12 (*para* or *p*)) are a class of commercially available and exceptionally stable 3D-aromatic boron-rich clusters that possess material-favorable properties such as thermal and chemical stability and high hydrophobicity.^32−37^ Carborane-based linkers in MOFs were first reported in 2007 at Northwestern University.^38^ The same group developed a family of thermally robust *p*-carborane-based MOFs over the following years,^39−45^ some of those showing high gas sorption capacities and good sieving behavior for gas mixtures. However, the use of the highly expensive and more symmetric *p*-carborane as a scaffold for ligand synthesis prevents any further research on potential real-world applications.^46^ Since 2016, some of us have pioneered the use of the cheaper *o*-^47,48^ and *m*-carborane^49−55^ derivatives with the primary objective of increasing the water stability of the prepared MOFs. We have demonstrated that introducing carborane moieties into MOFs can greatly enhance the framework’s water stability. The high hydrophobicity of some of these MOFs has provided them with high hydrolytic stabilities that allow their use in applications where water is always present. This is the case of the *m*-carborane-based [Cu_2_(*m*CB-L)_2_(DABCO)_0.5_(H_2_O)] (***m*****CB-MOF-1**; *m*CB-L = 1,7-di(4-carboxyphenyl)-1,7-dicarba-closo-dodecaborane; DABCO = 1,4-diazabicyclo[2.2.2]octane; Figure 1), which remains intact in water solutions under very harsh conditions.^52^

In this work, we have investigated our hydrophobic ultra-microporous *meta*-carborane-based MOF (***m*****CB-MOF-1**) for gas adsorption and CO_2_ separation applications. We show that ***m*****CB-MOF-1** exhibits unaffected performance for CO_2_:N_2_ separation under various humid conditions, validating the results by breakthrough dynamic separation experiments. Regeneration is also successfully achieved in mild conditions despite the presence of water, making this MOF a potential candidate for efficient CO_2_ separation from flue gas in industrial applications. The performance of our material is also compared with the well-known water-stable MOF-74(Ni) and shows that ***m*****CB-MOF-1** is a superior adsorbent for the separation of CO_2_:N_2_ mixtures under humid conditions.

## Results and Discussion

***m*****CB-MOF-1** shows a rigid twofold interpenetrated porous structure based on Cu_2_(OOC)_4_ paddle wheel units connected via four carborane-based dicarboxylate ligands, resulting in a square network. DABCO ligands connect to only one Cu atom of each paddle wheel, thus providing available Cu open sites (Figure 1).^52^ Such a structure differs from other related DABCO pillar-layered MOFs, such as [M_2_(1,4-bdc)_2_(DABCO)] (M = Ni, Zn; 1,4-H_2_bdc=1,4-benzenedicarboxylic acid),^56,57^ that do not contain open metal sites. The highly hydrophobic carborane moieties decorate the 1-D square MOF channels in ***m*****CB-MOF-1**, thus providing protection to the Cu_2_ paddle wheel units against hydrolysis or ligand displacement. Guest-free ***m*****CB-MOF-1**, denoted ***m*****CB-MOF-1′**, can be generated by heating at 120 °C in a vacuum and thus provide unsaturated Cu sites. As previously established, ***m*****CB-MOF-1′** shows negligible water sorption and has a Brunauer–Emmett–Teller (BET) surface area and pore volume of 756 m^2^ g^–1^ and 0.31 cm^3^/g, respectively, and it is also porous to CO_2_ (1.34 mmol g^–1^ at 313 K and 2 bar). It is stable in air for at least two years and submerged in 90 °C water for over two months.

**Figure 2:**
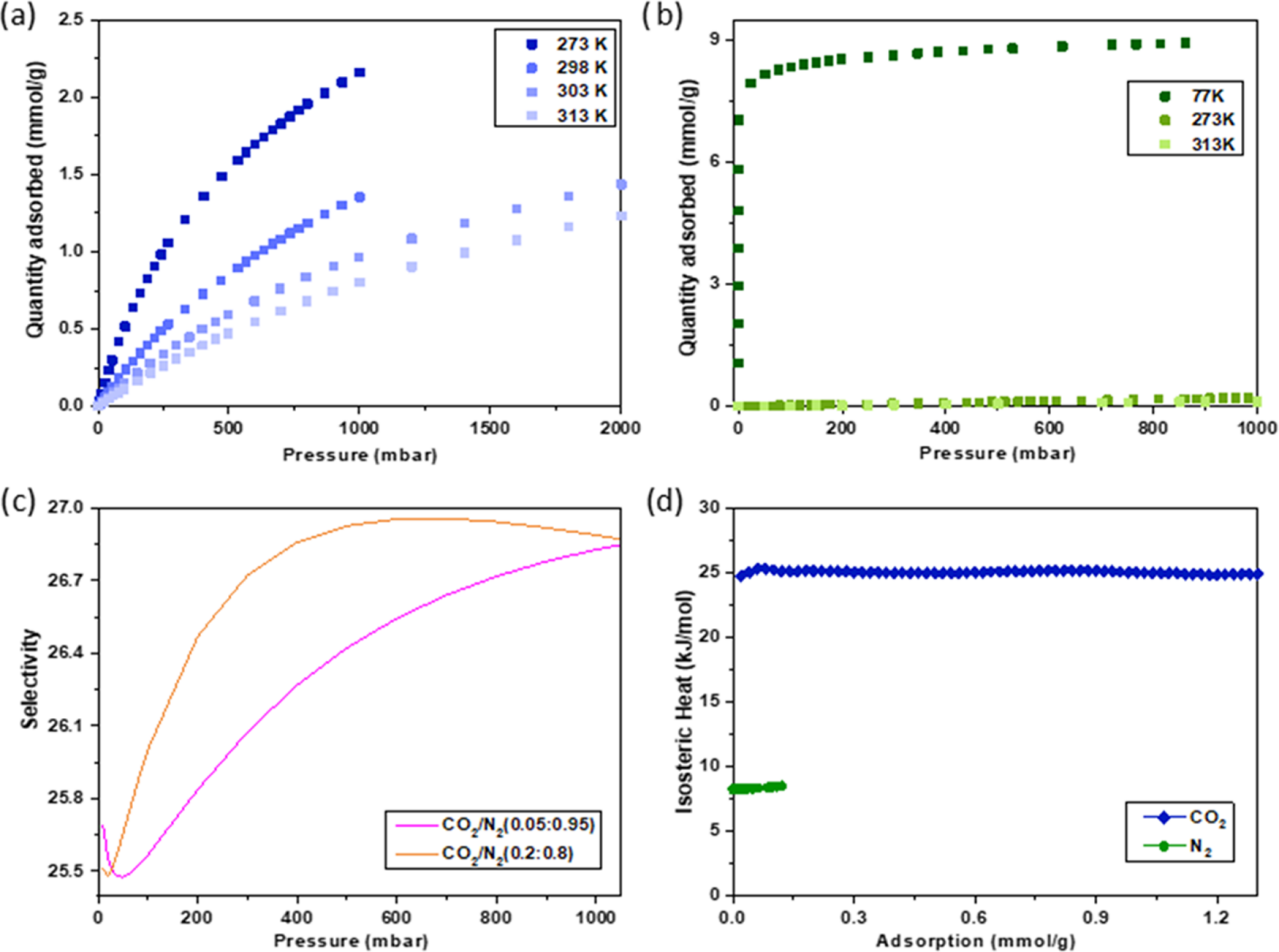
CO_2_ (a) and N_2_ (b) adsorption isotherms for ***m*****CB-MOF-1′** at various temperatures. (c) IAST-predicted selectivities for CO_2_:N_2_ (0.05:0.95 and 0.2:0.8, v/v) on ***m*****CB-MOF-1′** at 273 K. (d) Isosteric heats of adsorption for CO_2_ and N_2_ on ***m*****CB-MOF-1′**.

Although high hydrolytic stability is a prerequisite for applications where water is present, effective gas separation and good selectivities are not granted. Thus, we first evaluated the ability of ***m*CB-MOF-1′** to separate CO_2_ from N_2_ by single-gas isotherms measurements. Thus, CO_2_ and N_2_ sorption data at different temperatures and low pressure were collected (Figure 2a,b). At 1 bar, the CO_2_ uptakes on ***m*CB-MOF-1′** were 2.15, 1.35, 0.97, and 0.80 mmol/g at 273, 298, 303, and 313 K, respectively, these values being compatible with those of other ultra-microporous MOFs. When compared with N_2_ adsorption, the CO_2_ uptake was much higher at 273 K, as shown in Figure 2c, with the adsorption amount of 2.15 mmol/g for CO_2_ and 0.21 mmol/g for N_2_ at 1 bar at the same temperature. These results demonstrate that CO_2_ molecules have higher affinity with ***m*CB-MOF-1′** compared to N_2_, highlighting its advantage for highly effective separation of CO_2_ from N_2_.

To evaluate the evidenced adsorption affinity of the adsorbate–adsorbent, the isosteric heats of adsorption (*Q*_st_) for both gases were derived from the static isotherms at different temperatures on the basis of the virial method (see details in Experimental Section and SI). As shown in Figure 2d, the *Q*_st_ value of CO_2_ was in the range of 24.7–25.3 kJ/mol, while the value of *Q*_st_ for N2 was in the range of 8.2–8.5 kJ/mol, indicating a much stronger interaction between CO_2_ and ***m*****CB-MOF-1'**, compared to N_2_. The zero-coverage *Q*_st_ for CO_2_ was 24.7 kJ/mol, which is lower than that for the related Cu_2_-paddle-wheel-based MOF HKUST-1, showing also open Cu sites. The relatively low *Q*_st_ for CO_2_ suggests that the regeneration process of ***m*****CB-MOF-1'** would be easily achieved and with a low energy penalty.

Based on the adsorption performance difference for CO_2_ and N_2_, an ideal adsorbed solution theory (IAST) was adopted to predict the adsorption selectivity of theoretical CO_2_:N_2_ binary mixtures. The adsorption isotherms of CO_2_ and N_2_ were first fitted by dual-site Langmuir–Freundlich isotherm models (see details in Experimental Section and SI). The obtained fitting parameters are summarized in Table S1. It was noticed that both regression coefficients *R*^2^ were higher than 0.9999, indicating the excellent fitting of the data. Figure 2c shows the IAST selectivities at 273 K for CO_2_:N_2_ (with two different compositions: 0.05:0.95 and 0.2:0.8, v/v) in the pressure range 0–1 bar. Slightly lower CO_2_:N_2_ adsorption selectivities at lower pressures were observed. At 1 bar, the CO_2_:N_2_ adsorption selectivity at 273 K was in the range 25.5–27.0, with maximum values of 26.8 and 26.9 for the CO_2_:N_2_ ratios of 0.05:0.95 and 0.2:0.8, respectively. Nevertheless, there was no significant difference in the adsorption selectivities for the two ratios of gas mixtures. Overall, these results show that ***m*****CB-MOF-1'** may exhibit good separation performance of CO_2_ from N_2_.

**Figure 3:**
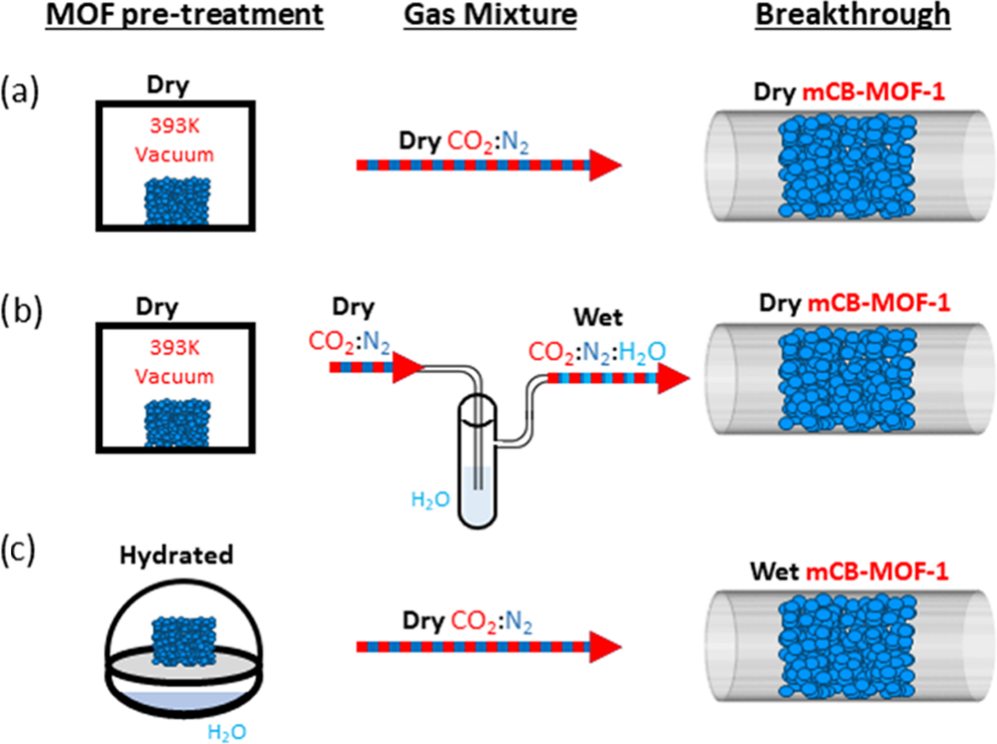
Schematic representation of MOF pretreatment (activated by heating under vacuum (a–c) and prehydrated under a water-saturated atmosphere for 24h (c)) and gas mixture composition (dry CO_2_:N_2_ (a and c), CO_2_:N_2_:H_2_O (b)) used in the breakthrough experiments.

Breakthrough measurements are commonly used to evaluate the potential of porous materials in gas separation processes in different conditions. CO_2_:N_2_ gas mixtures were passed through a fixed-bed column that was filled with 200 mg (average) of ***m*****CB-MOF-1'**. Breakthrough operation conditions ranged from 283 to 298 K, at 1 bar, in dry and humid conditions. The inlet mixture was set to a 15 mL min^–1^ flow of a dilution of CO_2_ in N_2_ (5, and 20%), and completed with an extra 1 mL min^–1^ of helium, used as a nonadsorbing trace. The use of a tracer is mandatory to assure the good performance of the measurement, setting time zero after it breaks through the column. In a typical experiment, the MOF sample was regenerated before the measurement at atmospheric temperature and pressure conditions of 15 mL min^–1^ Ar flow for 20 min. **Dry*****m*****CB-MOF-1** was activated at 393 K in a vacuum for 2 h. As moisture is a common pollutant in industrial gas flows, ***m*****CB-MOF-1** has been investigated under various conditions as shown in Figure 3.

**Figure 4:**
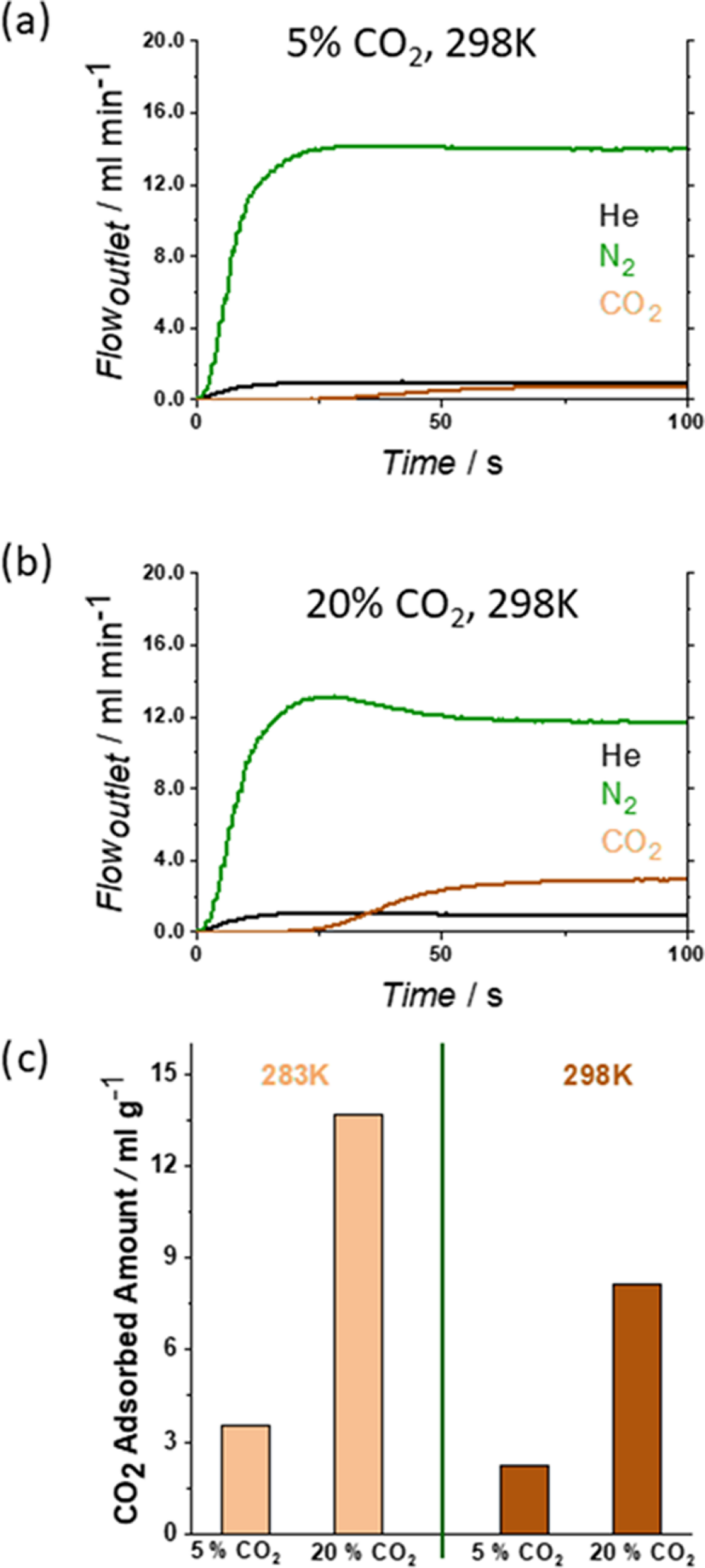
Breakthrough exit flow rates for **dry*****m*****CB-MOF-1′** at 298 K (5% CO_2_ (a); 20% CO_2_ (b)) and 1 bar. Time zero is set with the first detection of helium (tracer). (c) Amount of gas adsorbed on **dry*****m*****CB-MOF-1′** calculated from breakthrough profiles (a) and (b).

We first conducted CO_2_:N_2_ breakthrough experiments at two different CO_2_ dilutions (5 and 20%) and two temperatures (283 and 298 K) using **dry*****m*****CB-MOF-1′** (Figures 3a, 4a,b, S1, and Table S2) and a mass spectrometer to measure the outlet gas concentration (Figure 4c). The breakthrough curves indicate that ***m*****CB-MOF-1′** could effectively separate the two distinct CO_2_:N_2_ mixtures under ambient conditions. The N_2_ breakthrough occurred first and subsequently reached a plateau early. In comparison, the CO_2_ breakthrough time is significantly longer, confirming the effective separation performance of ***m*****CB-MOF-1′**. The typical roll-up effect especially appears at a high concentration (20% CO_2_, Figures 4b and S1b). CO_2_ selectivity is clearly observed also at the lower temperature of 283 K (Figure S1), as it also takes longer for this gas to breakthrough the column. As thermodynamically expected, a significant increase in the amount of adsorbed CO_2_ was observed at this temperature (Figure 4c and Table S2), a consequence of adsorption being an exothermic process. This remarkable selectivity (>1000; due to the negligible value of nitrogen adsorption), likely caused by the presence of open metal sites (OMS), makes ***m*****CB-MOF-1'** a promising material in CO_2_:N_2_ mixture separation. In addition, increasing the temperature, which favors the diffusion of the gases, causes a reduction in the CO_2_ capacity (see Figure 4c), but the selectivity remains very high. Thus, it is clear that the MOF acts as a molecular sieve, avoiding N_2_ adsorption and promoting CO_2_ capture in gas streams.

**Figure 5:**
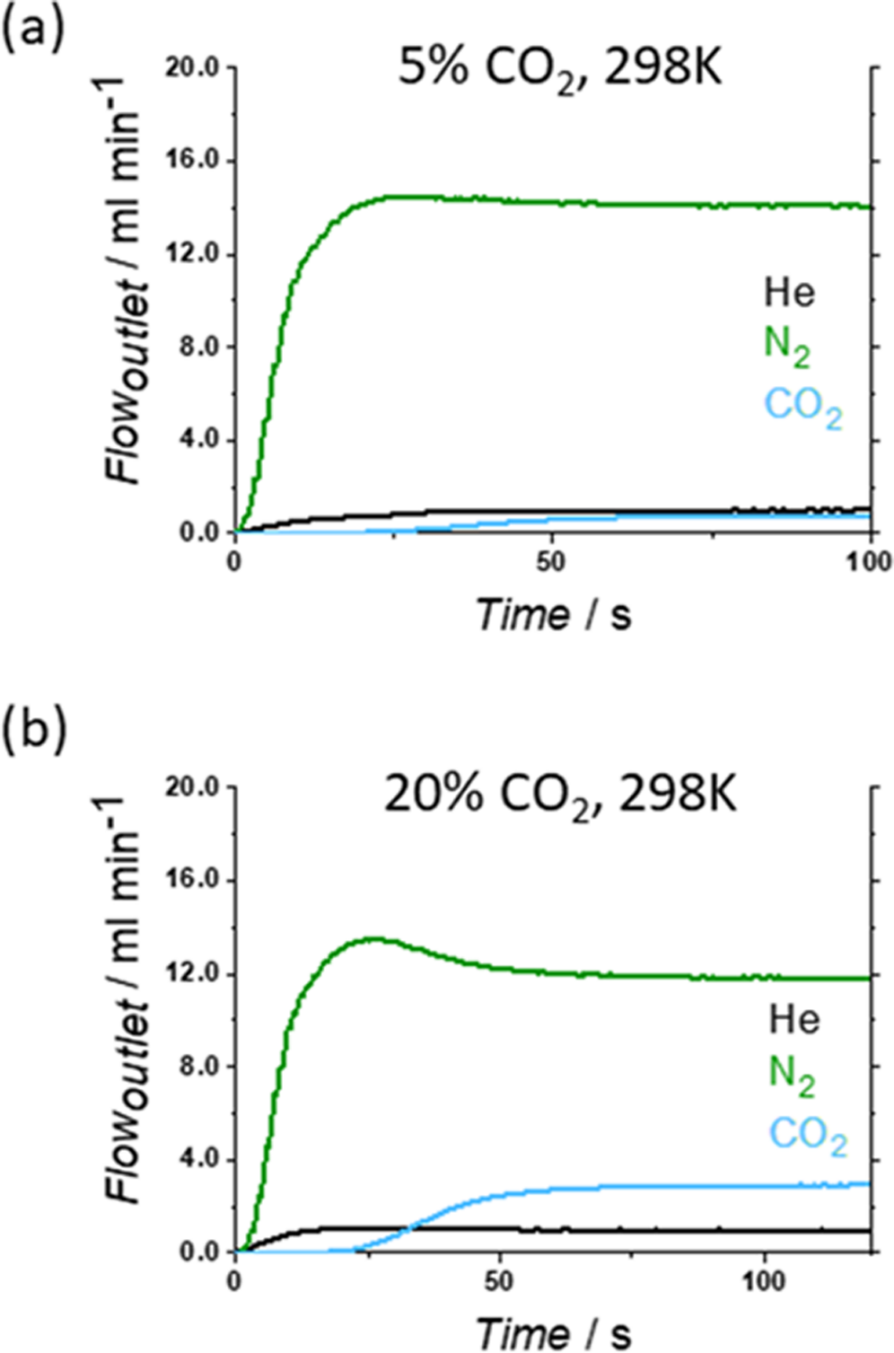
Breakthrough exit flow rates at 298 K and 1 bar on **hydrated*****m*****CB-MOF-1′**. Inlet composition corresponds to a 5% (a) or 20% (b) dilution of CO_2_ in nitrogen.

**Figure 6:**
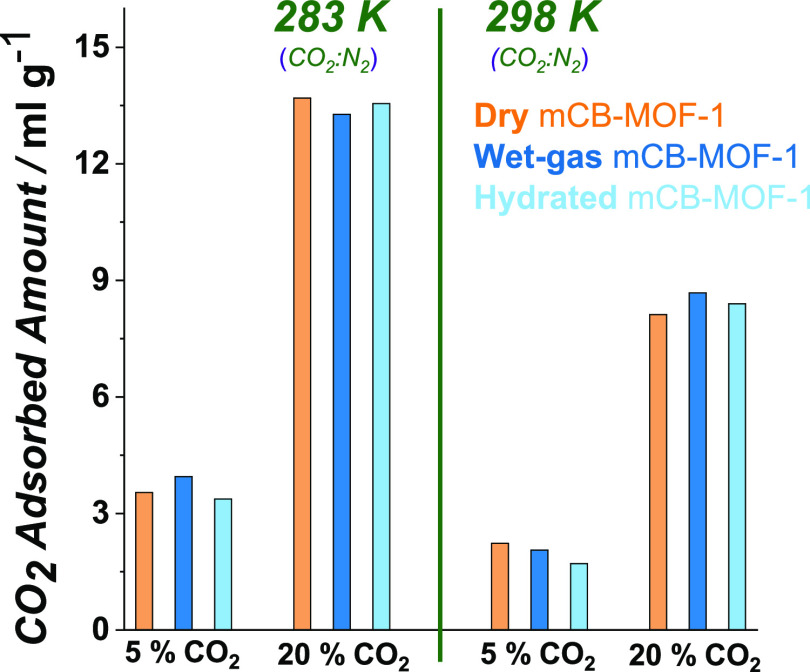
Amount of gas adsorbed on ***m*****CB-MOF-1'** (dry and humid conditions) calculated from breakthrough profiles at 1 bar (absolute pressure) for CO_2_:N_2_, at different concentrations (CO_2_: 5–20%), and different temperatures (283–298 K). Time zero is set with the first detection of helium (tracer).

The considerable CO_2_:N_2_ separation performance combined with the hydrophobicity of ***m*****CB-MOF-1'** prompted us to investigate the gas separation under high-humidity conditions. For that purpose, two different prehumidification steps were taken. (i) **Wet-gas mixtures**: CO_2_:N_2_ mixtures were passed through a bubbler containing room-temperature DI water (Figure 3b), assuring that ***m*CB-MOF-1′** was in permanent contact with a hydrated gas stream during the measurement. (ii) **Hydrated*m*CB-MOF-1′**: the sample was prehydrated under a water-saturated atmosphere (100% humidity) for 24 h and before the breakthrough experiments (Figure 3c). Whereas conditions in (i) are close to industrial gas flows, those in (ii) will pose a real challenge for this water-stable adsorbate selectivity, as, in the case of ***m*CB-MOF-1′**, the active sites are open Cu sites with high affinity for water molecules.^58^ Thus, we performed the dynamic breakthrough measurements under the same conditions as those for **dry*****m*****CB-MOF-1′** (5 and 20% CO_2_ dilutions; 283 and 298 K). The results (Figures 5, 6, S2, and S3 and Table S2) show that there are no significant differences in the amount of absorbed CO_2_ between the **dry**, **wet-gas**, or **hydrated*****m*****CB-MOF-1′**, evidencing the remarkable water resistance of the adsorbent and CO_2_:N_2_ separation performance even under high humidity (100% RH). Water is excluded from the pores due to the high hydrophobicity of the MOF channels. This leaves the open Cu sites available for the selective sorption of CO_2_ over N_2_.

Breakthrough curves and times of the **hydrated*****m*****CB-MOF-1′** (Figure 5) are nearly identical to those for dry ***m*****CB-MOF-1′** (Figure 4). These experimental results further demonstrate that water does not effectively enter the pores of our MOF nor affects the CO_2_:N_2_ separation selectivities during the breakthrough experiments. Effective separation and good selectivities were also obtained when using wet-gas mixtures (Figure S3). During the separation time, the outlet concentration of N_2_ is higher than 99%, indicating the outstanding separation for CO_2_:N_2_ even under humid conditions. Meanwhile, the CO_2_ longer break time confirms that the interaction between CO_2_ and ***m*****CB-MOF-1'** is stronger than that of N_2_. Additionally, the captured amount of CO_2_ was of the same order (Figure 6) when using wet-gas mixtures or **hydrated*****m*****CB-MOF-1′** or **dry*****m*****CB-MOF-1′**. Regeneration tests show that the separation performance was maintained after the adsorption–desorption cycles (Figure S4) and the crystalline phase of ***m*****CB-MOF-1′** was preserved after the separation process (Figure S5). These experimental results demonstrate that ***m*****CB-MOF-1** is a promising porous absorbent for CO_2_ separation over N_2_ for industrial processes.

**Figure 7:**
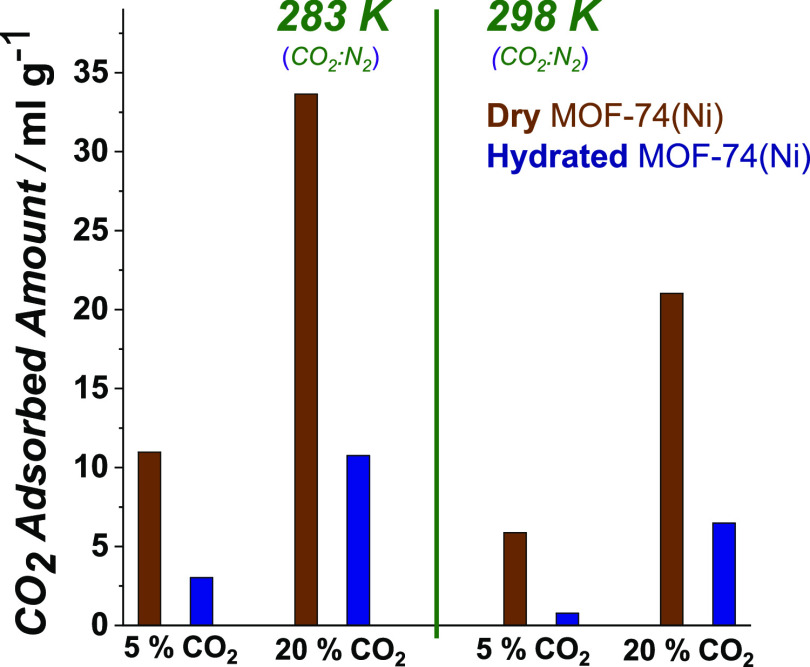
Amount of gas adsorbed on MOF-74(Ni) (dry and hydrated) calculated from breakthrough profiles at 1 bar (absolute pressure) for CO_2_:N_2_, at different concentrations (CO_2_: 5–20%), and different temperatures (283–298 K). Time zero is set with the first detection of helium (tracer).

Other MOFs with unsaturated metal centers in their structures, such as the MOF-74 family, are recognized as good candidates for the efficient postcombustion CO_2_ capture from water-containing flue gas generated from coal-fired power plants.^59,60^ From this family of MOFs, MOF-74(Ni) is one of the most water-stable ones.^61,62^ We have therefore selected MOF-74(Ni) as a reference material for a comparison study with our ***m*****CB-MOF-1′** and performed dynamic breakthrough experiments on dry and hydrated MOF-74(Ni) samples (Figure 3a,c, respectively). Although the MOF remains stable at these conditions, the results represented in Figure 7 clearly show the competitive adsorption of water in MOF-74(Ni). This resulted in a drastic decrease in the CO_2_ adsorption capacity and thus in its separation efficiency in the presence of water. The same trend is observed at all tested gas compositions and temperatures.

Regarding the related DABCO pillared MOFs [M_2_(1,4-bdc)_2_(DABCO)] (M = Ni, Zn), both materials show good CO_2_:N_2_ selectivities, but they collapse in a humid environment.^63^ Although these MOFs do not contain free coordination sites for water to readily interact with, they are not stable under >60% relative humidity at RT. Degradation seems to be related to water adsorption at defect sites in the MOFs.^64^ Considering water one of the most common contaminants, the possibility of performance in its presence is a relevant parameter for implementation in the current separation industry.

## Conclusions

This carborane-based material (***m*****CB-MOF-1**) presents not only competitive capacity and remarkable selectivity values for carbon dioxide adsorption in CO_2_:N_2_ mixtures, but it also stands for its excellent water stability along the separation process. The presence of unsaturated open metal sites explains the higher selectivity for CO_2_ sorption than that for N_2_. H_2_O is excluded from the pores due to the high hydrophobicity of our MOF.

After testing the adsorbent in different concentrations, temperatures, and humid conditions, two statements highlight the potential of this adsorbent: (i) the sieving effect derives in complete gas separation, achieving high CO_2_ capture efficiency, but allowing complete regeneration at mild conditions; and (ii) adsorption properties remain constant under moisture conditions, placing ***m*****CB-MOF-1** as an interesting alternative in separation processes involving an extreme humidity atmosphere. Given the mild affinity between gas and adsorbent ***m*****CB-MOF-1**, the gas separation with higher CO_2_ concentration at a lower temperature is specifically suitable for industrial purification processes over highly contaminated flows.

In addition, MOF-74(Ni) has been used as a reference due to its water stability properties; ***m*****CB-MOF-1** surpasses its performance and suppresses the rest of competitors in such challenging moisture operation conditions.

## Experimental section

### Materials

All chemicals were of reagent-grade quality. They were purchased from commercial sources and used as received. ***m*****CB-MOF-1** was synthesized as previously reported.^52^ Crystals of ***m*****CB-MOF-1** were immersed in acetone and exchanged once a day for three consecutive days, then filtered, and dried in air. The latter were then activated by heating at 120 °C under a dynamic vacuum for 2h.

### Characterization and Methods

Gas sorption–desorption measurements were performed using IGA001 and ASAP2020 surface area analyzers. The sample was first degassed at 130 °C for 12 h. Powder X-ray diffraction (PXRD) was recorded at room temperature on a Siemens D-5000 diffractometer with Cu Kα radiation (λ = 1.54056 Å, 45 kV, 35 mA, increment = 0.02°).

The isothermal parameters were well fitted by the double-site Langmuir–Freundlich (DSLF) method from the pure CO_2_ adsorption isotherms at 273 K. Fitting parameters of these equations as well as the correlation coefficients (*R*^2^) are listed in Table S1. Predicted selectivity for binary mixtures of CO_2_/N_2_ was analyzed using IAST.

### Ideal Adsorbed Solution Theory (IAST) selectivity

To investigate the separation efficiency of CO_2_:N_2_ mixtures for ***m*****CB-MOF-1**, the IAST method was used to predict the molar loadings at specific partial pressures using pure single-component isotherm fits.

For the adsorption isotherm of CO_2_ and N_2_ on ***m*****CB-MOF-1'** at 273 K, it was fitted with a double-site Langmuir–Freundlich (DSLF) model
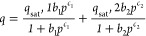
Here, *q*_sat,1_ and *q*_sat,2_ are saturation uptake (mmol/g) for sites 1 and 2, respectively; *b*_1_ and *b*_2_ are the affinity coefficients of sites 1 and 2, respectively; and *c*_1_ and *c*_2_ are the parameters for the deviations of an ideal homogeneous surface, respectively.

In the Ideal Adsorbed Solution Theory (IAST), two-gas adsorption selectivity could be calculated from single-component isotherm fitting parameters, defined as follows
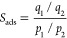


### Isosteric Heat of Adsorption

To evaluate the interactions between ***m*****CB-MOF-1** and these gas molecules (CO_2_ and N_2_), the isosteric heat of adsorption *Q*_st_ was calculated. In detail, *Q*_st_ was obtained by fitting adsorption isotherms of CO_2_ at 273, 298, and 303 K and N_2_ at 77 and 313 K with eq 1. Then, *Q*_st_ was calculated by eq 2.
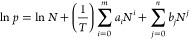
1
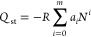
2where *p* and *N* are the pressure (Torr) and the quantity adsorbed (mg/g), respectively; *T* is the temperature (K); *a_i_* and *b_j_* are empirical parameters, respectively; *m* and *n* are the number of coefficients required to give a good fit to the isotherms, respectively; and *R* is the ideal gas constant (J·K^–1^·mol^–1^).

### Breakthrough Separation Experiments

Dynamic breakthrough experiments were done on an ABR (*HIDEN Isochema*) instrument. It is an automated breakthrough analyzer based on a fixed-bed adsorption column. In a typical experiment, pressure, temperature, and inlet composition are set and controlled. To determine the adsorption dynamic behavior of gas mixtures, the outlet flow composition is analyzed by an integrated mass spectrometer (HPR-20 *QIC*). The column was filled with 224 mg of ***m*****CB-MOF-1**. Before every measurement, the sample was regenerated at atmospheric temperature and pressure in 15 mL min^–1^ Ar flow for 20 min. Operation conditions ranged from 283 to 298 K at 1 bar. The inlet gases mixtures consisted of a 15 mL min^–1^ dilution of carbon dioxide in nitrogen (5–20% CO_2_ in N_2_). In all situations, gas mixtures resemble expected natural or industrial compositions. Time zero, in the analysis, is set with the first detection of helium due to its use as a tracer (1 mL min^–1^ of He in the feed flow). When using wet-gas mixtures, it was introduced as a moisture desiccator after the column to avoid any damage to the instrument or the mass spectrometer. Any effect derived from the introduction of these elements was corrected with a blank measurement.

## Supporting Information Available

Langmuir–Freundlich fitting parameters, breakthrough curves, experimental dynamic values, and XRD spectra (PDF)
