# Supplementary material for: Adsorptive Separation of CO2 by a Hydrophobic Carborane-Based Metal–Organic Framework under Humid Conditions
Source: ACS Appl Mater Interfaces. 2023 Jan 24;15(4):5309–16. doi: 10.1021/acsami.2c20373 (PMC9906620; doi:10.1021/acsami.2c20373)
Supplement: Supplementary file 1 — am2c20373_si_001.pdf [file am2c20373_si_001.pdf]

Supporting Information

Adsorptive separation of CO_2 by a Hydrophobic Carborane-Based Metal-Organic Framework under Humid Conditions

Lei Gan, [†‡] Eduardo Andres-Garcia,[†&] Guillermo Mínguez Espallargas, [&*] José Giner Planas[‡*]

[‡] Institut de Ciència de Materials de Barcelona (ICMAB-CSIC), Campus UAB, 08193 Bellaterra, Spain.
[&] Instituto de Ciencia Molecular (ICMol), Universidad de Valencia, c/Catedrático José Beltrán, 2, 46980 Paterna, Spain.
† Equal contribution.
[*]Emails: guillermo.minguez@uv.es, jginerplanas@icmab.es

Table S1. Double-site Langmuir-Freundlich fitting parameters of CO_2 and N_2 adsorption isotherm on ***m*CB-MOF-1'** at 273 K.

***m*CB-MOF-1'**	$q_{sat,1}$ (mmol/g)	b_1 (KPa^{-1})	c_1	$q_{sat,2}$ (mmol/g)	b_2 (KPa^{-1})	c_2	**Adj. R^2**
CO_2	2.671066	0.004237	1.024780	1.753189	0.029466	1.001424	0.9999975
N_2	3.575533	0.000524	1.012954	0.033866	0.014131	1.034506	0.9999865

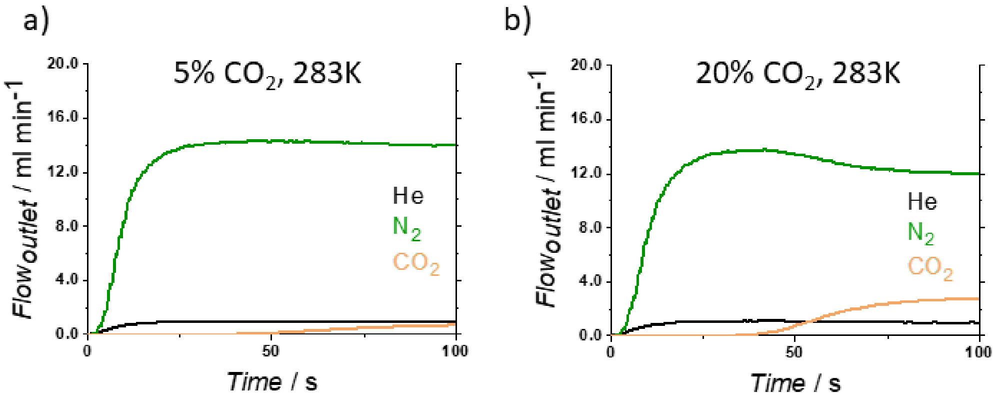

Figure S1. Breakthrough exit flowrates vs. time at 283 K and 1 bar, on **dry *m*CB-MOF-1'**. Inlet composition corresponds to a 5 % (a) or 20% (b) dilution of CO_2 in nitrogen. Time zero is set with the first detection of helium (tracer).

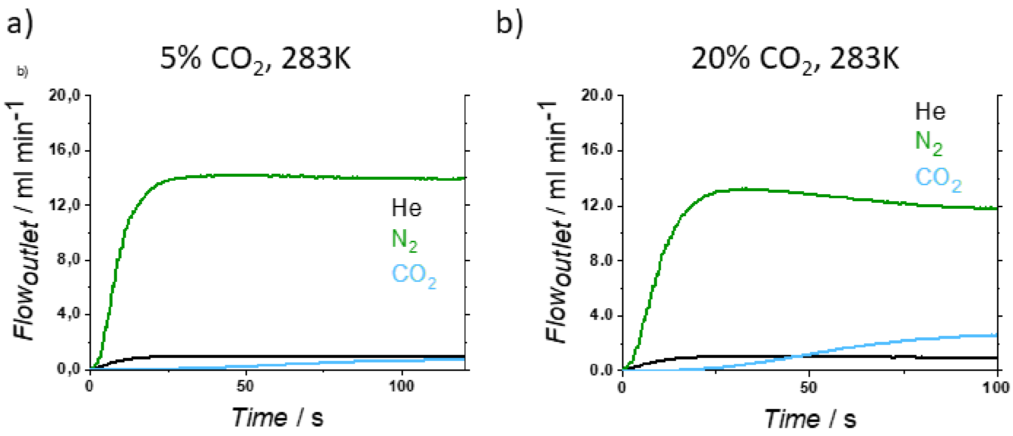

Figure S2. Breakthrough exit flowrates vs. time at 283 K and 1 bar, on **hydrated *m*CB-MOF-1'**. Inlet composition corresponds to a 5 % (a) or 20% (b) dilution of CO_2 in nitrogen. Time zero is set with the first detection of helium (tracer).

Table S2. Experimental dynamic values from the integration of the breakthrough curves: carbon dioxide adsorbed amount (ml g^{-1}) for ***m*CB-MOF-1'** (dry, wet-gas or hydrated). See Figure 3 for experimental setup.

**dry *m*CB-MOF-1'**	5% CO_2 (CO_2:N_2)	20% CO_2 (CO_2:N_2)
283 K	3.5	13.7
298 K	2.2	8.1
**Wet-gas**	5% CO_2 (CO_2:N_2)	20% CO_2 (CO_2:N_2)
283 K	3.9	13.3
298 K	2.1	8.7
**hydrated *m*CB-MOF-1'**	5% CO_2 (CO_2:N_2)	20% CO_2 (CO_2:N_2)
283 K	3.4	13.6
298 K	1.7	8.4

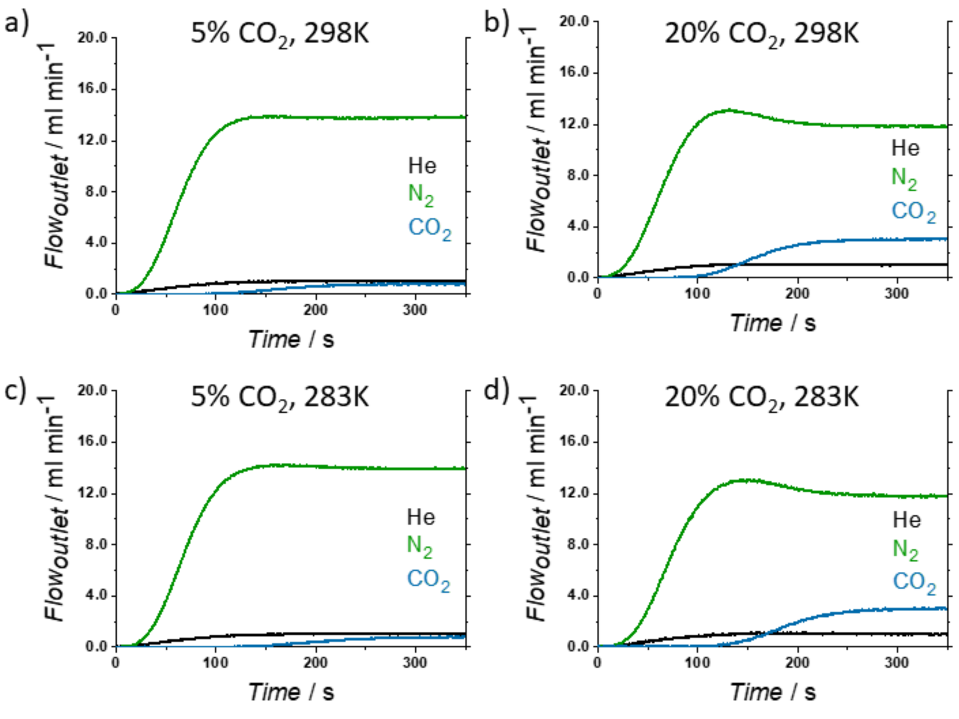

Figure S3. Breakthrough exit flowrates of **wet-gas mixtures** vs. time at 298K or 283 K and 1 bar, on ***m*CB-MOF-1'**. Inlet composition corresponds to a 5 % (a and c) or 20% (b and d) dilution of CO_2 in nitrogen. Time zero is set with the first detection of helium (tracer).

For the experiments using wet-gas mixtures (See Figure 3), a desiccator was place between the breakthrough column and the Mass spectrometer. This was crucial to

protect the spectrometer. This particular set up affected the breakthrough curves and times, so that they seems longer that in the other experiments in this work. The effect has been corrected so that a correct comparsion can be done. To calculate adsorption values, flow areas were integrated above the breakthough profiles. In order to apply a practical correction for the dessicator effects, blank measurements (without MOF sample in the column) were performed to evaluate its impact in the gas profiles.

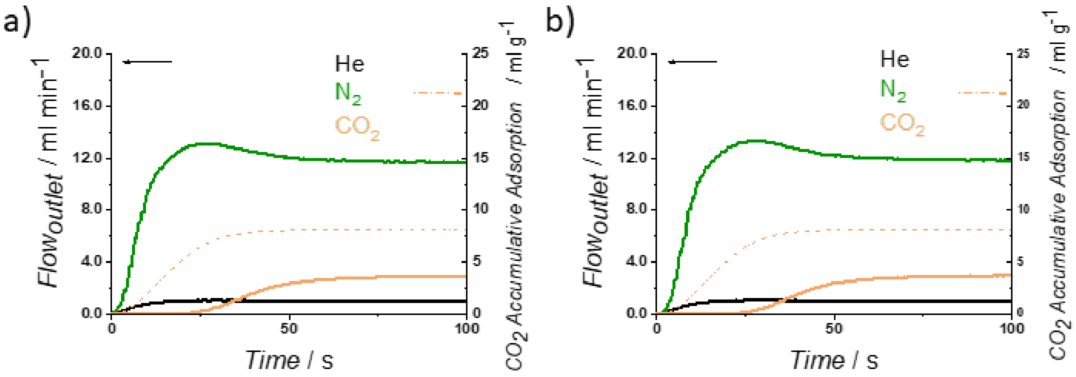

Figure S4. Breakthrough exit flowrates vs. time at 298 K and 1 bar, on **dry *m*CB-MOF-1'**. Inlet composition corresponds to a 20 % dilution of CO_2 in nitrogen: (*a*) original measurement; and (*b*) replica measurement. Time zero is set with the first detection of helium (tracer).

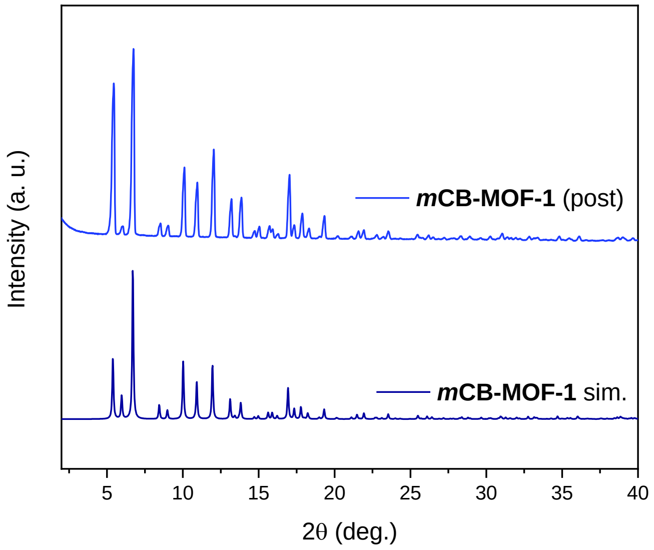

Figure S5. mCB-MOF-1 simulated (bottom) and experimental (top) powder X-ray diffraction after breakthrough experiments.

REFERENCES

(1) Gan, L.; Chidambaram, A.; Fonquernie, P. G.; Light, M. E.; Choquesillo-Lazarte, D.; Huang, H.; Solano, E.; Fraile, J.; Viñas, C.; Teixidor, F.; Navarro, J. A. R.; Stylianou, K. C.; Planas, J. G. A Highly Water-Stable Meta -Carborane-Based Copper Metal–Organic Framework for Efficient High-Temperature Butanol Separation. *J. Am. Chem. Soc.* **2020**, *142* (18), 8299–8311. https://doi.org/10.1021/jacs.0c01008.
